# Paul Ehrlich and Almroth Wright

**Published:** 1991-12

**Authors:** William Gillespie


**REFERENCES — see p.118**

### Paul Ehrlich and Almroth Wright

**William Gillespie, MD, FRCP**

Early in the 20th century both were considered very great men. Ehrlich is still so regarded, but Wright's reputation is much diminished.

**Paul Ehrlich** (1854-1915) was Jewish, born in Silesia, qualified M.D. Leipsig. He became director of the Institute for Experimental Therapy, Frankfurt (1899-1915). He was among the greatest scientific workers in medicine since Pasteur.[1,2] Ehrlich had a deep understanding of organic chemistry. He wrote "Mine is a kind of visual, 3-dimensional chemistry. Benzene rings and structural formulae disport themselves in space before my eyes. It is this faculty that has been of supreme value to me".[2]

Ehrlich's days were filled with an excited, enthusiastic concentration on his scientific ideas and plans for research. He made three great contributions:

(1) Invented modern cytological staining methods based on chemical affinities betwen dyes and cell structures;
(2) His studies of Immunology won him a Nobel prize in 1908;
(3) He invented scientific chemotherapy.

In well-planned research he and his assistants synthesized hundreds of organic arsenical compounds, starting from Atoxyl and tested them in rabbits infected with spirochaetes. The 606th agent to be tested was Arsphenamine ("Salvarsan"), remarkably successful against syphilis and the standard treatment until penicillin came. Ehrlich called it a "Magic Bullet" which destroyed the germs without harming the host.

On a personal note, I knew Professor Hans Sachs, formerly an assistant of Ehrlich, who came to Dublin as a refugee in 1939. He and Frau Sachs told me many stories of the great man. Often, enthusiastic about a new idea, he would take a cab and keep it waiting during a lengthy visit to a colleague. This made him popular with the cab drivers of Frankfurt. I took Sachs to see the film "The Magic Bullet" in which Edward G. Robinson played Ehrlich. Sachs was delighted and said it accurately portrayed Ehrlich, his enthusiasm and method of working. The producer must have been advised by someone who knew him well.

**Sir Almroth Wright** (1861-1947), son of an Irish clergyman and Swedish mother studied languages and medicine simultaneously at Trinity College, Dublin, with excellent degrees in both. He went immediately into physiological research in Leipsig, Cambridge, Sydney. He became Professor of Pathology at the Army Medical College, Netley, in 1892, and Professor of Pathology, St. Mary's Hospital, Paddington in 1902. At Netley he embarked on Bacteriology. His study of prophylactic anti-typhoid vaccination, which he did not invent, and which was a controversial subject, led to the wide use of the vaccine, saving thousands of lives during the Great War. Another of his important contributions was in the treatment of war wounds at the military hospital in Boulogne (1914-1918). He opposed the treatment of wounds with strong antiseptics, which damaged leucocytes. He insisted that wounds be cleaned and excised as soon as possible, in France, and not left for operation until after evacuation to England. Hence, although he had never been a house surgeon, Wright greatly improved wound surgery.

Wright's success with prophylactic vaccination led him to believe that existing infections could be cured by injecting therapeutic autogenous vaccines made from the causative organism. The timing of injections was regulated by repeated estimations of the phagocytic power of the blood (opsonic index). The need for repeated blood samples led Wright to develop micro-techniques, using tiny volumes of capillary blood, described in his book "The Technique of the Teat and Capillary Pipette".

Vaccine therapy was once regarded the most important of Wright's contributions and for many years dominated the treatment of many infectious diseases — and yet it was valueless. His definitive paper in the Lancet[3], described in detail a few men with boils and acne, who improved during treatment with autogenous vaccine; but there were no controls! Foster[2] says that this paper was one of the most influential in medical bacteriology — even though there are few more worthless papers in scientific literature!

And yet — the idea of stimulating the body's defences to promote recovery from infection was a good one. Conceivably, it might return in a more sophisticated form, e.g. in treating H.I.V. infection . Who knows?

The collapse of vaccine therapy greatly reduced Wright's reputation — unfairly, perhaps. Vaccine therapy had positive, indirect results. It stimulated the development of clinical bacteriology. Colebrook[4] gives a sympathetic picture of Wright's Inoculation Department at St. Mary's where he and his assistants worked long hours, seeing patients with all sorts of infections, making diagnostic cultures and preparing vaccines. One assistant, Alexander Fleming, discovered penicillin.

Wright had many distinguished visitors, including Ehrlich. A non-medical visitor, George Bernard Shaw, based the central character of "The Doctor's Dilemma" on Wright.

Wright was very intelligent, very cultured, an accomplished linguist. He wrote two books on philosophical subjects. His weakness was a lack of judgement, and over-confidence in the ability of an intelligent man to know, by instinct, when he was right. He mistrusted statisticians and said that every commonsense man is capable of forming a judgement as to whether or not a particular result is the result of the operation of chance!

Wright's capacity to make wrong judgements was shown by his position in a contemporary political controversy, women's suffrage. He opposed it vigorously. He did not dislike women but believed that they lacked judgement. In fact, Ehrlich and Wright were linked by a woman. After Ehrlich died, his secretary, Martha Marquardt, spent her life, collecting records of his career and writing his biography. Later, over 30 years after Ehrlich's death, Wright invited her to London and arranged financial support for her to write an English version.[5] In this book she acknowledged the help of "the late Sir Almroth Wright, for more than fifty years a friend of Ehrlich".

**REFERENCES — SEE P.118**

**ctd. from p.107 ''Ehrlich and Wright'' — Gillespie**

**REFERENCES**

1. W. BULLOCH. A History of Bacteriology; Oxford, 1938.
2. W. D. FOSTER. A History of Medical Bacteriology and Immunology; London, 1970.
3. A. E. WRIGHT. Notes on the Treatment of Furunculosis, Sycosis and Acne by the Inoculation of a Staphylococcus Vaccine. *Lancet.* **1.** 1902, 874-884.
4. L. COLEBROOK, Almroth Wright, Provocative Doctor and Thinker; Wm. Heinemann, London, 1954.
5. MARTHA MARQUARDT. Paul Ehrlich (with introduction by Sir Henry Dale). Wm. Heinemann, London, 1949.

